# Micro-Doppler Based Classification of Human Aquatic Activities via Transfer Learning of Convolutional Neural Networks

**DOI:** 10.3390/s16121990

**Published:** 2016-11-24

**Authors:** Jinhee Park, Rios Jesus Javier, Taesup Moon, Youngwook Kim

**Affiliations:** 1 Department of Information and Communication Engineering, Daegu-Gyeongbuk Institute of Science and Technology (DGIST), Daegu 42988, Korea; pjhdrm@dgist.ac.kr; 2 School of Electronics Technology, ITT Technical Institute, Clovis, CA 93612, USA; JRiosRamos@itt-tech.edu; 3 Department of Electrical and Computer Engineering, Lyles College of Engineerning, California State University, Fresno, CA 93740, USA; youngkim@csufresno.edu

**Keywords:** radar, micro-Doppler signatures, aquatic activity classification, convolutional neural networks, transfer learning

## Abstract

Accurate classification of human aquatic activities using radar has a variety of potential applications such as rescue operations and border patrols. Nevertheless, the classification of activities on *water* using radar has not been extensively studied, unlike the case on dry ground, due to its unique challenge. Namely, not only is the radar cross section of a human on water small, but the micro-Doppler signatures are much noisier due to water drops and waves. In this paper, we first investigate whether discriminative signatures could be obtained for activities on water through a simulation study. Then, we show how we can effectively achieve high classification accuracy by applying deep convolutional neural networks (DCNN) directly to the spectrogram of real measurement data. From the five-fold cross-validation on our dataset, which consists of five aquatic activities, we report that the conventional feature-based scheme only achieves an accuracy of 45.1%. In contrast, the DCNN trained using only the collected data attains 66.7%, and the *transfer learned* DCNN, which takes a DCNN pre-trained on a RGB image dataset and fine-tunes the parameters using the collected data, achieves a much higher 80.3%, which is a significant performance boost.

## 1. Introduction

Increased demand for security, law enforcement, rescue operations, and health care has accelerated research in the detection, monitoring, and classification of human activities [1,2] based on remote sensing technologies. In particular, the unique micro-Doppler signatures from human activities enabled diverse and extensive research on human detection and activity classification/analysis using radar sensors [3,4,5,6,7,8,9,10,11,12]. More specifically, the authors of [6] extracted direct micro-Doppler features such as bandwidth and Doppler period, the authors of [7] applied linear predictive code coefficients, and the authors of [8] applied minimum divergence approaches for robust classification under a low signal-to-noise ratio environment. Furthermore, the authors of [9] suggested to use particle filters to extract features, the authors of [10] employed biceptrum-based features, the authors of [11] utilized orthogonal pseudo-Zernike polynomials, and features based on the centroid or the singular value decomposition (SVD) have been exploited in [12]. Compared to optical sensors, the electromagnetic radar sensors can operate in all weather conditions, regardless of lighting changes, and hence are competitive for the applications that require robust operation. So far, however, most of the research has focused on the classifications of human activities on dry ground.

In addition to dry ground, the accurate classification of human activities on water (namely, *the aquatic activities*) has wide applications in rescue operations or coastal border patrols; for example, as monitoring human activities on ocean at night or on a foggy day using optical sensors can be extremely challenging, and the robust detection and classification using radar becomes desirable. However, for the activities on water, it becomes more difficult to design informative handcrafted features based on micro-Doppler signatures, as in [6]. The reason is because human motions on water tend to be more irregular than those on the dry ground, and the micro-Doppler signatures become noisier due to water drops and waves. Moreover, the radar cross section (RCS) of parts of a human subject on water is low so that the Doppler signatures become less apparent than those on dry ground. Therefore, collecting large-scale training data of high quality, which is crucial for the application of machine learning algorithms, has become more difficult and expensive.

In this paper, we investigate whether the micro-Doppler signatures can be still utilized to the more challenging case of classifying human activities on water. First, we carry out a simulation study on the micro-Doppler signatures of swimming activities using the point scatterer model to understand whether the signatures for different activities can be discriminative. Then, we continue our preliminary study in [13] by applying deep convolutional neural network (DCNN) directly to the spectrogram for the classification of human activities on water. As has been widely proven in many applications [14,15,16,17], the motivation of applying the DCNN is clear: instead of handcrafting the features for a given classification task, the DCNN can automatically learn the features as well as the classification boundaries directly from the two-dimensional (2-D) spectrogram data. We show that the DCNN becomes much more powerful, particularly with the transfer learning technique, for situations in which collecting high-quality data and devising handcrafted features are more challenging, as in the case of classifying human activities on water.

Applying deep neural networks (also known as *deep learning*) to the micro-Doppler signature-based classification has been attempted only recently. Namely, the authors of [17] were the first to apply a DCNN to the micro-Doppler signature-based human detection and activity classification, the authors of [18] utilized stacked auto-encoder for fall motion detection, and the authors of [19] applied a DCNN similar to that in [17] but with a limited dataset. To the best of our knowledge, leveraging the transfer learning of a DCNN has not been attempted before for the micro-Doppler signature-based activity classification.

For our experiments, we used Doppler radar and collected spectrogram data of five human subjects performing five different activities on water: freestyle, backstroke, and breaststroke swimming, swimming while pulling a floating boat, and rowing. We implemented two versions of the DCNN and compared their performances with a baseline Support Vector Machine (SVM) that implements the handcrafted features in [6]. The first DCNN is the one trained from scratch using the collected spectrogram data, which exactly follows the approach of [13,17]. The second DCNN is the transfer learned DCNN, namely, we take a pre-trained DCNN, which is trained on a separate, large-scale RGB image classification dataset, ImageNet [20], and fine-tune the network parameters using the collected spectrogram data. Our result of the transfer learned DCNN significantly outperforming other schemes illustrates that the features learned by the DCNN for the RGB image classification can be successfully transferred to the micro-Doppler signature-based classification. In the following sections, we summarize our simulation study and data collection process, explain the DCNN training in more detail, and present the experimental results.

## 2. Micro-Doppler Simulation of Swimming Activities

It is an interesting research question whether it is possible to obtain meaningful micro-Doppler signatures for the human activities on water when a subject is illuminated by radar. To that end, we carried out a simulation study of micro-Doppler signatures for the swimming activities to understand their characteristics before collecting real measurement data. When a person is swimming, the major detectable parts of a human body from radar are arms. Hence, if the arm motion of a person is properly modeled, we can simulate the expected micro-Doppler signatures as similar works were done for human walking in [3,4]. In this section, we focus on two swimming styles, the freestyle and backstroke, and simulate the micro-Doppler to verify whether discriminative signatures could be obtained.

**Figure 1:**
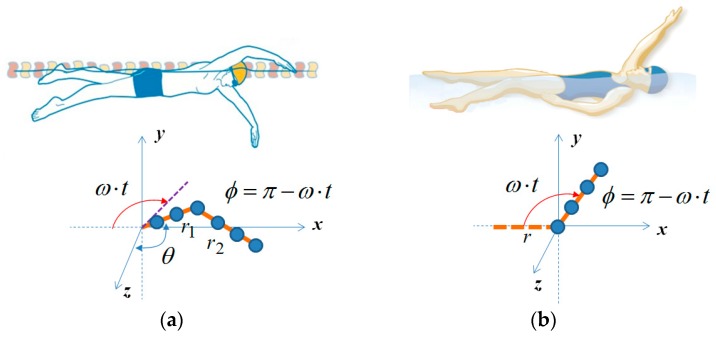
Modeling of arm motions of (**a**) freestyle and (**b**) backstroke.

**Figure 2:**
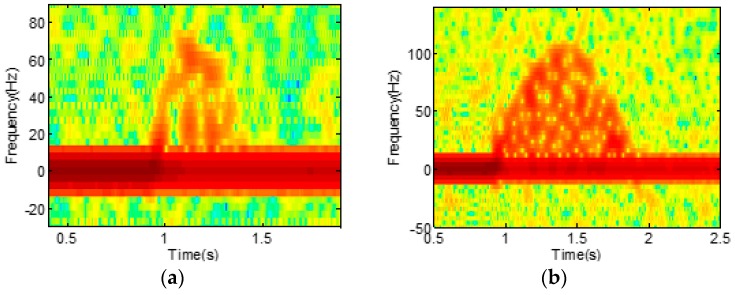
Simulated spectrograms of (**a**) freestyle and (**b**) backstroke.

Based on [21], we calculated the velocity of point scatterers of upper and lower arms of a swimmer for each swimming style. The arms are modeled as a sum of point scatterers with a separation of wave length (λ), and we assumed the received signal becomes the linear superposition of Doppler shifts from all point scatters. For simplicity, a single scattering model is employed while ignoring multiple reflections. For the freestyle, we modeled the motion as two rotating cylinders, in which the upper arm (r_1_) rotates with the angular velocity of ω while keeping θ constant as shown in Figure 1a, and the lower arm (r_2_) is assumed to be always on the x–z plane. In this case, the velocity of each point scatters can be analytically calculated through trigonometry. We set r_1_ as 0.28 m, r_2_ as 0.42 m, and ω as 2π rad/s. With an operating frequency of 7.25 GHz and a sampling rate of 1 Ksps, the simulated spectrogram with additional Gaussian noise is presented in Figure 2a. For the backstroke, in contrast, we assumed the motion as a single rotating cylinder as shown in Figure 1b. We set the length (r) of the cylinder as 0.7 m and the angular velocity, ω, as π rad/s, since the rotation of the backstroke is typically slower than the freestyle. The resulting simulated spectrogram is shown in Figure 2b.

By comparing Figure 2a,b, we observe clear sinusoidal signatures in both figures. However, we also see that the signatures from the freestyle and backstroke are not identical and show a subtle difference. Such a difference, which is confirmed by the real measurement data in the next section, suggests that the micro-Doppler signatures for the activities on water can indeed be discriminative, and a powerful classifier may be necessary for the accurate classification of the activities.

## 3. Measurements of Human Activities on Water

For the measurement of the five activities on water, we used the same setup in [13] and collected the spectrogram data of the activities of five human subjects in a swimming pool. The average height and weight of human subjects are 178 cm and 76 kg. The activities include freestyle, backstroke, and breaststroke swimming, pulling a floating object, and rowing a small boat. As we focused only on the human signatures on water, the measurement data was collected in a more controlled environment than that of a sea or a lake. Doppler radar, which operated at 7.25 GHz with an output power of 15 dBm, was used to capture human motions as each human subject approached the radar system. We used vertical polarization assuming that human motion, especially arm motion, effectively interacts with illuminated electromagnetic (EM) waves.

**Figure 3:**
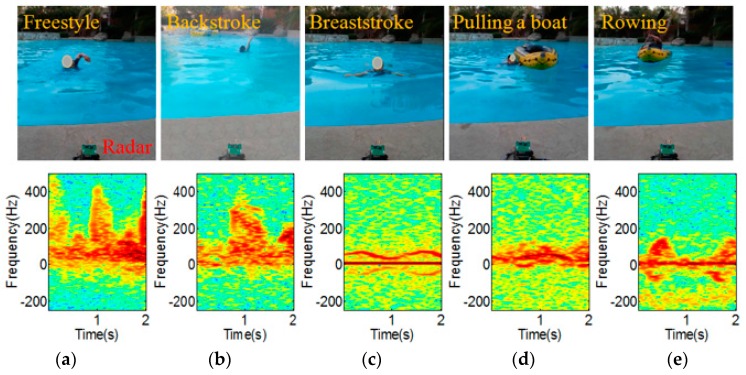
Example pictures and spectrograms of (**a**) Freestyle; (**b**) Backstroke; (**c**) Breaststroke; (**d**) A swimming person pulling a floating boat; and (**e**) Rowing.

The received signal was processed with the joint-time frequency analysis to investigate its time-varying micro-Doppler characteristics. In the short-term Fourier transform, the fast Fourier transform (FFT) size was set at 256, and the non-overlapping step size at 20 ms. Example pictures and spectrograms for each activity are presented in Figure 3. While we recognize that each activity indeed possesses unique micro-Doppler signatures, as suggested by the simulation study in Section 2, they are not as clear as those from dry-ground measurements because of the low RCS and interference of water waves and drops.

In order to construct the training and test data sets for our DCNN-based approach, we measured a single human subject five times for each activity. From each measurement, we randomly extracted five spectrograms with 2 s intervals (100 pixels), potentially overlapping with each other. In the cropped spectrogram, the Doppler frequency was between 0 Hz and 500 Hz (256 pixels). The negative frequency does not contain significant information because the human subject was approaching the radar during the measurement. As a result, we have a total of 625 data samples (i.e., spectrograms), which consist of five actions with 25 samples for each action for every 5 subjects. The dimension of each spectrogram was 252 (frequency) by 100 (time).

## 4. Classification Method Using Deep Convolutional Neural Network (DCNNs)

### 4.1. DCNN Trained from Scratch

Recently, DCNNs are revolutionizing many applications that mainly involve 2-D data, e.g., image recognition. The key reason is due to their power of automatically learning hierarchical representations (i.e., features) for given classification tasks directly from the raw data input. Such a revolution was realized due to the explosion of data, the advent of high-performance computing processors such as the graphic processing unit (GPU), and continued algorithmic innovations. A more thorough overview on DCNNs and deep learning in general can be found in [22], and the references therein.

The authors of [17] were the first to apply a DCNN to micro-Doppler signature-based human activity classification by casting the problem as an image classification problem. Applying a DCNN to micro-Doppler signature directly achieved the accuracy essentially in par with the handcrafted feature-based state-of-the-art scheme in [6]. In order to apply the framework of [17] to the classification of human activities on water, we can simply feed the spectrogram data obtained in Section 3 and train the parameters of the DCNN. Regarding the handcrafted feature-based scheme, however, we observe that the micro-Doppler signatures of the activities on water are more subtle compared to those of the activities on dry ground, as can be seen in Figure 3; hence, it is not clear whether the handcrafted features developed in [6] would also lead to high accuracy when classifying the activities on water.

**Figure 4:**
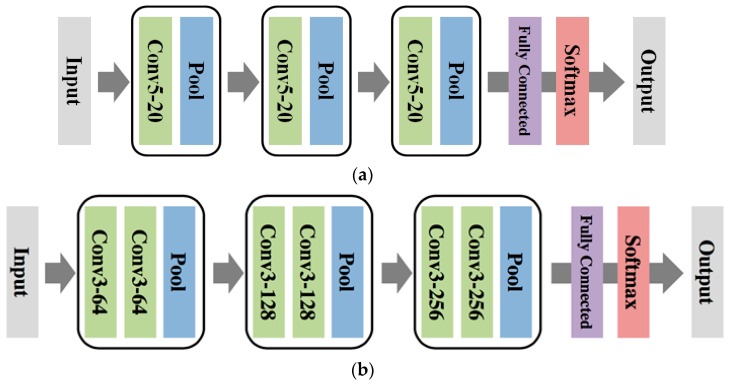
The architectures of the DCNN learned from scratch. (**a**) DCNN-Scratch-I and (**b**) DCNN-Scratch-II.

We tried two different DCNN configurations. The first model (DCNN-Scratch-I), depicted in Figure 4a, is identical to the one considered in [17]. That is, as shown in the figure, we used three convolution layers, in which each layer had 20 convolution filters with 5 pixels-by-5 pixels in size, respectively, followed by a Rectified Linear Unit (ReLU) activation function and a 2 pixels-by-2 pixels max pooling layer. We used 500 hidden nodes with ReLU activation for the fully connected layer, followed by a softmax classifier. The network has about 4 million parameters. The second configuration (DCNN-Scratch-II) is inspired by the recent advances in the DCNN architectures [23,24] that use consecutive convolution filters before pooling as depicted in Figure 4b. The number of filters and filter sizes for each layer are given in the figure, and the network has about 55 million parameters. To train both models, we used the mini-batch Stochastic Gradient Descent (SGD) with momentum, with the learning rate 0.01 for DCNN-Scratch-I and 0.0005 for DCNN-Scratch-II, the momentum 0.9, and the batch size of 50. Dropout was used at the fully connected layer with a rate of 0.5, and the maximum iteration of the mini-batch SGD update was 5000. We also used zero padding at the boundary of the data.

### 4.2. Transfer Learned DCNN

While the DCNN trained from scratch with the collected spectrogram could learn useful features and achieve high classification accuracy as in [17], the small amount of our training data for the activities on water (i.e., 625 samples) may not realize the full potential of the DCNN. Therefore, we also experimented with the transfer learned DCNN.

Transfer learning [25] generally refers to the techniques that transfer the knowledge or models learned from a certain task to some other related, but different, task (i.e., a target task) that typically lacks sufficient training data. Such techniques commonly improve the accuracy of the target task provided that the two tasks possess some similarity in the data distribution. While various transfer learning techniques exist, the transfer learning of the DCNN can be done with the following simple procedure: Take a DCNN that is already trained for some classification task that is related to the target task and possesses a large amount of training data, replace the output classification (softmax) layer that matches the target task, and fine-tune (i.e., update) the DCNN parameters with the limited amount of training data from the target task.

By following the above procedure, we take a DCNN that is pre-trained with the ImageNet dataset and fine-tune the network parameters using the spectrogram data collected for the activities on water. ImageNet [20] is a large-scale benchmark dataset that consists of 1.5 million RGB training images that are 224 pixels-by-224 pixels, created for computer vision tasks such as image classification or object detection. The dataset was used for the annual ImageNet Large-Scale Visual Recognition Challenge (ILSVRC) and significantly accelerated the innovation for the DCNN-based algorithms. Furthermore, the ImageNet pre-trained DCNN has been successfully used as a base model for transfer learning to some other applications (i.e., target tasks) such as style classification [26] or earth observation classification [27] that have limited training data. However, most of the transfer learning schemes regarding fine-tuning the ImageNet pre-trained DCNN were applied to target tasks that still take the RGB images as input. Therefore, since the characteristics of the natural RGB images in ImageNet and the spectrograms collected from the Doppler radar are completely different, it is not apparent at all whether our approach, i.e., the transfer learning of the ImageNet pre-trained DCNN to the spectrogram-based classification of activities, can be effective.

**Figure 5:**
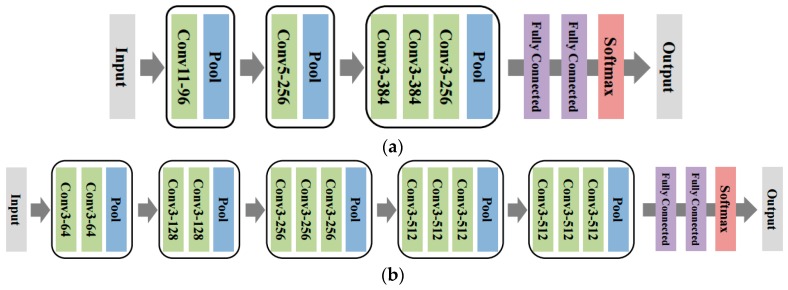
The architectures of DCNN used for transfer learning. (**a**) AlexNet and (**b**) VGG16.

In our experiments, we show such effectiveness of transfer learning with two seminal DCNN models pre-trained on ImageNet, namely, AlexNet [15] and VGG16 [23]. AlexNet, as is depicted in Figure 5a, has five convolutional layers and three fully connected layer with about 60 million parameters. The model is the winner of the 2012 ILSVRC challenge [15] and became the catalyst of recent research on the DCNN. Since the spectrogram image has a single channel, we simply copied the data for each of the three input channels for AlexNet. For fine-tuning the network parameters, the final softmax layer of AlexNet was replaced with a new softmax layer that has five classes, and the entire network parameters were updated with the spectrogram data. The architecture of the second base model, VGG16, is given in Figure 5b. As can be seen in the figure, VGG16 has a much deeper architecture than the others, i.e., 13 convolutional layers and 3 fully connected layers. The network has 138 million parameters and achieved about half the error rate on the ImageNet test set compared to AlexNet in 2014 ILSVRC [23]. We follow the same procedure of transfer learning for VGG16 as to that of AlexNet. We call the two transfer learned DCNNs with each base model as DCNN-TL-AlexNet and DCNN-TL-VGG16, respectively. The hyper-parameters for the mini-batch SGD training of both models were identical to those of the DCNNs trained from scratch, except for learning rates of 0.001 for AlexNet and 0.0005 for VGG16.

## 5. Experimental Results

We followed the approach of [17] and carried out five-fold cross validation (CV) using the spectrogram data collected as in Section 3 to evaluate the performances of the compared methods. Each fold consists of data from each human subject (i.e., 125 samples); thus, the classification accuracy measures the generalization abilities of the algorithms across the human subjects. Note that the preliminary study in [13] carried out the five-fold CV using only the data from a single subject, which is why the accuracy was much higher. For the DCNN models, since the model configurations (e.g., the number of layers and the number of convolution filters) were fixed as explained in Section 4, the only hyper-parameter we chose via CV was the early stopping parameter; namely, we picked the SGD iteration that gave the best average test score. We used Caffe [28] to implement the DCNN and utilized the Intel Xeon processor E5-2620-v3 and NVIDIA GTX Titan X GPU for our experiments.

Before we applied the DCNN, we implemented eight handcrafted features from the spectrograms similar to the ones developed in [6] and applied the SVM as a baseline conventional method. The features include torso Doppler, Doppler bandwidth, Doppler offset, the bandwidth without Doppler, the Doppler periodicity, and the variance of the Doppler energy distribution. Note these features extract some general information on micro-Doppler signatures and are not just specifically designed features for dry ground activities. We note that, due to the poor quality of the spectrograms for the activities on water, a few features could not be calculated occasionally; hence, we replaced a missing feature with the average value of the corresponding feature for the same activity and for the same person. For the SVM, we used the Gaussian kernel and chose the best parameters for the kernel width and the regularization parameter for the slack variables among 2500 combinations via CV.

Table 1 summarizes the CV results. We see that the baseline SVM that utilizes the handcrafted features achieves an accuracy of 45.1%. While it is certainly better than a random guess among the five activities (i.e., 20%), we can clearly see that the handcrafted features developed for the activities on dry ground [17] are not generalizing enough to the activities on water. On the contrary, we observe that the DCNN-Scratch-I and DCNN-Scratch-II achieve accuracies of 61.9% and 66.7%, respectively, which are significantly better than the baseline SVM (a 40% improvement). This result proves the robust nature of the DCNN for micro-Doppler signature-based classification; namely, instead of designing a separate set of features for different classification tasks, the DCNN can directly learn the features from the raw data of the new task and achieve high accuracy. Furthermore, we see that the transfer learned models, DCNN-TL-AlexNet and DCNN-TL-VGG16, achieve 74.6% and 80.3%, respectively, which are again significantly better than the DCNN models learned from scratch. Comparing to the baseline SVM, DCNN-TL-VGG16 is 78% more accurate. From this result, we observe that the features learned by the DCNN for RGB image classification can be extremely useful even when transferred to the micro-Doppler signature-based human activity classification problem that has only a limited number of spectrogram data for training. The reason is because the DCNN learns features in a hierarchical way; hence, the low-level features learned for RGB image classification, such as edge or texture detectors, may be utilized and fine-tuned to detect useful micro-Doppler signatures for the classification.

**Figure 6:**
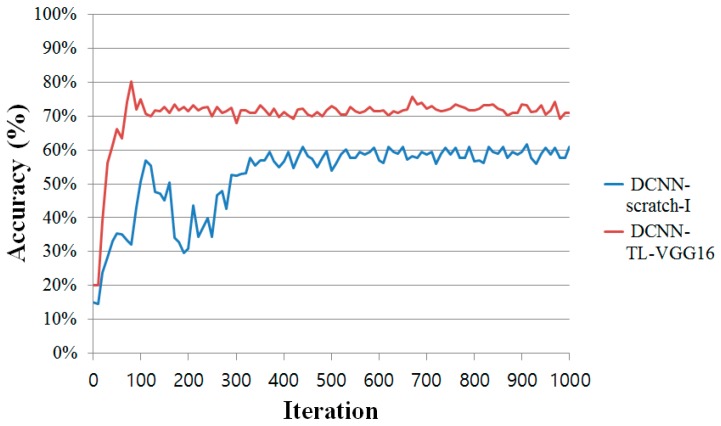
Average learning curves of DCNN-Scratch-I and DCNN-TL-VGG16. The horizontal axis stands for the number of mini-batch SGD update iterations.

Figure 6 shows the learning curves (averaged over the 5 folds) of the two DCNN models, DCNN-Scratch-I and DCNN-TL-VGG16. From the figure, we observe that the DCNN-TL-VGG16 consistently dominates DCNN-Scratch-I with a significant gap in accuracy and converges quickly to attain the best accuracy in around 100 iterations of the SGD updates. On the contrary, DCNN-Scratch-I, which learns the DCNN parameters from scratch, needs more iterations (around 400 iterations) to converge to the best accuracy it can achieve. From this result, we clearly see that the transfer learning of the DCNN can be done very efficiently and effectively.

**Figure 7:**
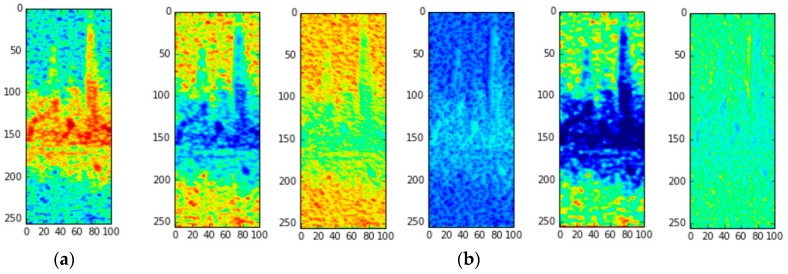
Visualization of feature maps. (**a**) Raw spectrogram for “freestyle”; (**b**) Visualization of 5 feature maps of DCNN-TL-VGG16 after the first convolution layers.

In Figure 7, we provide the visualization of a sample input spectrogram data as it passes through the convolution layer of DCNN-TL-VGG16, the best model. Figure 7a is the raw spectrogram input for one of the “freestyle” motions, and Figure 7b is the visualization of the feature maps (i.e., the results of the convolution of each filter) after the first convolution layer. While there are many filters in the first layer, we only visualized the feature maps that showed the most contrasting characteristics. As stated in Section 3, the micro-Doppler signatures of the “freestyle” motions are quite noisy, so the handcrafted features in [6] may not be able to discriminate the activity well from others. However, as we can see in Figure 7b, the convolution filters in DCNN-TL-VGG16 can successfully capture various aspects of the input spectrogram, e.g., textures and edges, such that the high classification accuracy can be achieved using those captured features as shown in our experiments.

## 6. Discussion and Conclusions

In this paper, we considered the problem of classifying human activities on water based on micro-Doppler signatures. We first carried out a simulation study suggesting that the classification in such a scenario can be challenging. Then, with real measurement data, we applied several DCNN-based methods and achieved almost double the accuracy of the baseline SVM that uses handcrafted features developed for the activities on dry ground. Our contributions are as follows: (i) We carried out the initial, rigorous work on the classification of human aquatic activities, which can be applied to several applications; (ii) We showed the robust nature of the DCNN-based classification framework for the micro-Doppler signature-based activity classification; (iii) We showed that the transfer learning of the ImageNet pre-trained DCNN can be extremely useful when there are only a small number of Doppler radar-based spectrogram data. This result shows that the DCNN approach and the use of the transfer learning technique are promising for further extension on micro-Doppler signature-based detection and classification problems.

As mentioned in the introduction, human activity classification in the ocean would be one of the most important applications of this study. It should be noted, however, that, when ocean waves are very strong, it becomes very difficult to accurately detect and classify a human activity. Ocean waves consist of several components such as breaking waves, resonant waves, capillary waves, and gravity waves, and they produce different kinds of scatterings, e.g., Bragg scattering, burst scattering, and whitecap scattering. Thus, when the waves are strong, such complex scatterings as well as the large RCS of the waves make it difficult to detect human signatures through radar. However, when the waves are less strong, such as those from lakes, the micro-Doppler signatures from a human subject can still be identified and used, and a more systematic study for such a situation constitutes a direction of potential future research.

In addition, another fruitful future research direction is a comparison of the performance of a DCNN and all combinations of existing feature-based schemes [6,7,8,9,10,11,12] to investigate the optimum performing methods for micro-Doppler signature-based human activity classification. 

## Figures and Tables

**Table 1:** Five-fold cross validation results for the compared schemes.

	Fold 1	Fold 2	Fold 3	Fold 4	Fold 5	Average
**SVM**	27.2%	36.0%	56.8%	70.4%	35.2%	**45.1%**
**DCNN-Scratch-I**	64.3%	49.8%	92.5%	49.8%	53.2%	**61.9%**
**DCNN-Scratch-II**	68.0%	51.2%	98.4%	63.2%	52.8%	**66.7%**
**DCNN-TL-AlexNet**	60.0%	79.2%	93.6%	69.6%	70.4%	**74.6%**
**DCNN-TL-VGG16**	70.4%	72.0%	99.2%	82.4%	77.6%	**80.3%**
